# Rethinking the time spent at school: Could flexibility improve engagement and performance for students and teachers?

**DOI:** 10.1007/s11125-023-09638-9

**Published:** 2023-05-03

**Authors:** Jim Watterston, Yong Zhao

**Affiliations:** 1 Melbourne Graduate School of Education, University of Melbourne, Grattan Street, Parkville, VIC 3010 Australia; 2 School of Education and Human Sciences, University of Kansas, Lawrence, USA

**Keywords:** School time, Learning time, Education innovations, School reforms, COVID-19, Four- day school week

## Abstract

Is it possible to reduce the time students spend in classrooms and schools? Would such a reduction be better for learning and retaining teachers? How should learning be more flexibly enacted in the post-pandemic era? This article discusses the possibilities of rethinking school participation and calls for schools to reconsider the necessity and costs/benefits of forcing students and teachers to be physically present in schools for the traditional 5 days a week.

The traditional construct of time spent on-site at school is one of the most stubborn and perhaps neglected concepts when thinking about how to optimize students’ learning. Every school system has established that students must be in school with a teacher for a certain number of hours and days during a year; thus, both students and teachers must be present physically in school for the specified number of days and hours. The Covid-19 pandemic disrupted this tradition for a short period of time, but by and large, schools immediately reverted back to the “old normal” of having students and teachers attend schools for 5 full days a week.

During the height of the pandemic, in nearly all countries across the world, for varying periods of time, students and their teachers engaged in remote learning during stages of lockdown, before returning to the traditional 5-day-a-week industrial education model of face-to-face learning at desks in classrooms. This normal 5-day school week, however, has become increasingly problematic for the education profession post-Covid, which brought with it enormous challenges, along with some innovative opportunities to rethink the way we enact our work and live our lives. As we have moved beyond harsh lockdown restrictions to manage Covid-19 variants, many countries and school systems have reported that a great number of employees have resigned from their teaching jobs and fewer students are undertaking teacher education courses, as many seek more flexible employment opportunities that allow them to work from home or in remote locations.

Employment for millions of people has been customized, and employees are advantaged by less time spent commuting; more comfortable home offices; and an ability to work anywhere, anytime. The education profession, however, has been challenged to identify and create opportunities for employee and student flexibility, and consequently many school leaders have identified this lack of customization as the reason many teachers are choosing to seek employment elsewhere.

The potential for changing the basic construct of schooling for the better is staring us all in the face, but most of us can’t see it or haven’t considered using this lever to explore the opportunities and outcomes it may bring. Such a rethink is initiated by asking (a) if there would be any benefit for students, families, and staff if we reconsidered whether 5 days per week in classrooms is the most advantageous construct and (b) whether there is a more flexible way of engaging students, while providing more learner agency at the same time.

For most people, these questions will never be asked, because schools have always been 5 days a week, and the time commitment has rarely ever been challenged. Prior to Covid-19, most parents worked at least 5 days, from Monday through Friday, so school attendance was therefore aligned with parental requirements to be at their place of employment. Our experience during the pandemic has demonstrated that education and the world of work do not necessarily have to be totally on-site or enacted during the traditional hours 5 days a week.

## The 4-day school week

In pre-pandemic times, some variation occurred in the traditional 5 days that the overwhelming number of students and teachers were required to attend their school. In some countries, students went to school for up to 6.5 days. There were also calls for increasing school time based on the assumption that students would learn more. For example, one report called for significantly increasing the amount of school time for students in the United States (The National Education Commission on Time & Learning, 1994).

A group of U.S. schools has implemented a 4-day school week (Armitage, 2022; Kilburn et al., 2021; Thompson & Ward, 2022). The number of school districts that offer 4 school days has grown significantly over the past 3 decades, from mere hundreds in the 1990s to over 1600 in more than 20 states in the United States in recent years (Kilburn et al., 2021). Some models of the 4-day school week add more instructional time to each of the days to minimize the reduction of total school time, although not all schools lengthen the 4 days.

Virtually all the districts that switched to 4 days are in rural areas or small towns. The original cause of this switch from 5 to 4 days was largely financial and related to staffing, although research shows that the actual financial saving for a district is less than 2% (Kilburn et al., 2021). The results for some schools have been quite positive in terms of reactions of parents, students, and staff, who have overwhelmingly welcomed the change. The impact on academic outcomes, however, is not clear, with some analyses showing little or no negative impact, while others identified more significant negative effects over a longer time (Anderson & Walker, 2015; Barshay, 2022; Sawchuk, 2021; Thompson & Ward, 2022).

The 4-day school week is no doubt a significant change in education. It has been, however, unfathomable for the majority of schools to even consider the possibility of a different attendance construct. Indeed, some U.S. states have attempted to block schools from moving to this model and insisted schools offer at least the required number of school hours. Schools that have switched to 4 days per week have also had to deal with numerous legal and practical issues.

## Changing the time spent in school may be more significant than we think

Surprisingly, minimal attention has been given to the significance of a change in the time spent educating students in general. Current discussions and research have been largely focused on the outcomes of the change in the 1600 rural districts. Little attention has been given to the implications of changes driven by the quest for better student engagement and improved teacher satisfaction, rather than changes driven simply by a district’s attempt to save money.

The switch from 5 to 4 days a week requires a big change in mindset. It challenges the inexorable tradition of running schools for at least 5 days a week. As we move into a post-Covid world, we should explore the potential for all schools to consider changing school attendance requirements as a viable option for better educational engagement, improved student outcomes, and flexible work practices.

Such a switch challenges the notion of schooling, although traditional research on the 4-day school week did not necessarily analyze this perspective. The traditional belief is that students must spend a certain amount of time in school, which actually means in class, where everybody is technically learning the same thing that has been prescribed, directed, and managed by the teacher. This model has been actively challenged prior to and during the pandemic for many reasons, such as diversity in students’ capabilities and needs, as well as the questioned value of homogenous teaching (McDiarmid & Zhao, 2022; Zhao, 2018b). Many have advocated for classroom differentiation (Tomlinson, 2014), but not many have considered and explored undoing classes or school time.

The world, and education in particular, changed during the pandemic. Education during this time amplified problems of practice that were challenging for decades (Zhao & Watterston, 2021). We cannot afford to return to the old normal now that we have seen students prosper while working remotely, while others disengaged completely, just as teachers performed similarly. There has never been a more important time to contemporize our education systems than now. Just as so many employees are moving to jobs that allow them to work anytime, anywhere, our next generations of problem solvers and leaders should be provided with similar ways of learning and working.

## What is learning?

One construct of a change to compulsory school attendance could be to maintain a 5-day week but allow at least one of those days at home for more senior students. If students were to have a fifth day of learning on their own, what would happen? A day out of the school could be supported by their teachers providing guided opportunities for reading, research, study, taking online courses, doing math homework, or working on group projects with other learners. They could also, of course, do all of these on this day, with or without being prescribed, predetermined, and directly managed by teachers.

Is this learning? Does a student always have to learn what schools and teachers want them to learn or teach them? Could this fifth day be about discovery and exploration of civic issues and a pursuit of personal passions and interests, which could be shared back at school? If such learning on their own does not improve the test scores on school subjects, does it count as learning or is it a distraction? If the students develop capabilities such as resilience, curiosity, communication, collaboration, independence, self-direction, socialization, and perseverance through their own independent work outside school, is it good learning?

Answers to these questions depend largely on one’s belief about learning. If one follows the tradition that only school subjects are worth learning and only test scores matter, the answers to all questions would be no. But if one has a transformed view of learning and believes learning is about much more than school subjects, the answers may be different.

The transformed view of education has been advocated for a long time (Tyack & Cuban, 1995). Many have questioned the merits or lack thereof of one-size-fits-all education (Barber et al., 2012; McDiarmid & Zhao, 2022; Wagner, 2008) and proposed new possibilities (Watterston & Zhao, 2020, 2021; Zhao & Watterston, 2021), but schools have largely remained the same, as has the assessment of learning.

## Breaking down the tradition

The 4-day school week could be the beginning of a significant breakdown of traditional schooling. It enables us to rethink the most fundamental and symbolic element of schooling. If students only need to attend their school for 4 days a week, do all of those days need to be spent in the traditional classroom? More importantly, if learning is reconstructed so it is directed by students having agency over their learning (Wehmeyer & Zhao, 2020; Zhao, 2012, 2016b), how school days are organized should be revised anyway.

In many ways, Covid-19 education experiences provided opportunities similar to what we propose for the 4-day school week. The Covid-19 pandemic forced virtually all schools to close for days and months, and learning was placed online (Zhao, 2020b). In these forced global educational experiments, some schools offered online learning that differed from what the majority of schools offered, which essentially replicated school days. Innovative schools did not ask their teachers to teach online the same way as they did in person. Instead, they allowed teachers to adjust their teaching and empowered students to have more autonomy over their learning. These students had at least some ownership of their own learning and appeared to have engaged with the online learning more than in-person learning (Fleming, 2020), although the majority of emergency online learning during Covid-19 has not been praised by many parents, teachers, and students.

Indeed, many students during lockdown periods were not able to thrive in the online environment without a teacher overseeing and directing the work and time management. A number of school leaders in Australia have estimated that, for those students who were provided with a work schedule by their teacher at the beginning of the day and then asked to report back their progress at the end of the day, around 20% were unable complete any of the set tasks (Watterston & Zhao, 2021). It would seem that the traditional didactic classroom management approach of teachers in pre-Covid times may have prevented many students from having agency over their work during the challenging times of Covid-19.

Could the notion of enhancing the learner agency of older students be promoted by working on their passions and projects anytime, anywhere, through a shorter school week with options for self-directed learning outside the school gates? Covid-19 learning experiences should be reflected upon more than simply focusing on the so-called learning loss (Zhao, 2021a), which has attracted the most attention in education policy and practice. One of the major focus points should be the diversity of practices in different schools, especially those schools that have moved away from a simple replication of traditional practices. Higher education institutions, such as Harvard, MIT, and Stanford, have all examined and paid attention to the innovations of education and deviations of student life during the pandemic and developed lessons for change in the future (Harvard Future of Teaching & Learning Task Force, 2022; MIT Ad hoc Committee on Leveraging Best Practices from Remote Teaching for On-Campus Education, 2022; Stanford Digital Education, 2022). In K-12 education, however, such reviews and examinations seem to be less comprehensive, if they have been done at all. Upon deeper reflection, explication, and experimentation, we did find numerous innovations enabling students to break away from prescribed curriculum and teaching to explore learning of their own interests, in their own time.

The 4-day school week could allow schools to declutter their curriculum, which corresponds with an emerging trend in education that is calling for schools to teach less but more deeply (Hamilton et al., in press; Reich, 2022). Teachers could be employed for 5 days a week, with at least 1 flexible day provided to work remotely on elements such as preparation, marking, reporting, and research.

## Rethinking school time and learning

Constructive changes to the traditional 5-day school week thus should not be considered simply a practical or financial solution. Coupled with the experiences during the pandemic, we can reimagine different time arrangements for schools, but with the same amount of investment and connection. There are, however, a few points to consider.

First, learning time is not the same as school time. Students are able to learn without being in the physical classroom or school (Zhao, 2021b). Motivated and challenged students may not need to be in schools 5 or more days a week.

Second, students should be able to learn without being taught and managed by a teacher all the time. Teachers, of course, are important and necessary, but they do not need to be watching, supervising, and teaching all students all the time. Students should be provided with agency over their learning from the early years so that engagement is heightened and becomes a catalyst to learning productively on their own or in groups. Teachers should be able to rethink their roles to focus more on guiding, facilitating, supporting, and intervening (Zhao, 2018a, 2022). Teachers and schools should reconsider how much instruction must be given, and how much time should be managed and directed by students.

Third, learning is much more than mastering school subjects (Watterston & Zhao, 2021; Zhao & Watterston, 2021). Much has been said about the importance of skills in thinking, communicating, collaborating, and problem identifying and solving, as well as personal traits such as resilience, curiosity, creativity, and confidence (Duckworth & Yeager, 2015; Trilling & Fadel, 2009; Wagner, 2012; Zhao et al., 2019). Students’ social and emotional health and capacities have also been increasingly recognized as significant outcomes of learning, despite the controversy around social and emotional learning (SEL; see CASEL, 2019; Jones et al., 2017; Zhao, 2020a). These skills are difficult to teach in the traditional classroom, as they are much deeper than knowledge. Such skills need authentic and meaningful experiences to develop; thus, we need to provide students with more autonomy and time to explore and expand their learning experiences on their own and with the support of adults, as necessary.

Fourth, personalization of learning has emerged as one of the important directions of educational enhancement. To meet the massively diverse needs of students, to address the persistent educational inequity, and to develop the individual strengths of each student (Zhao, 2016a, 2018b, 2019), it is fundamental that we personalize learning to empower students to customize their learning experiences. Personalization requires schools to be flexible with curriculum, time, assessment, and facilities. More importantly, it requires students to be self-directed and empowered so they are interested in and capable of driving and directing their learning.

## Summary

The direction of education is at a critical phase at this juncture. With decades of calls for major reforms, the outcomes and quality of education have not demonstrated a holistic improvement, largely because the reforms have not directly addressed the problem of individuality and the students’ desire to pursue meaningful and engaging learning experiences. Today, with the rapid development of technology, significant disruptions and innovations brought about by the Covid-19 pandemic, an uncertain future created by climate changes, more pandemics, human conflicts, geopolitical reconfiguration, and reshaping of globalization, education needs to and is able to make the necessary changes to make learning strength based and passion driven.

In this context, the redesign of the traditional 5-day school week is an opportunity that should be seriously considered by all schools and systems, not simply as a way to save money but more as a way to personalize education by providing flexibility for both teachers and students to transact their work more productively.
